# Genomic characterization and antibiotic susceptibility of biofilm-forming *Borrelia afzelii* and *Borrelia garinii* from patients with erythema migrans

**DOI:** 10.3389/fcimb.2025.1619660

**Published:** 2025-07-07

**Authors:** Giorgia Fabrizio, Ilaria Cavallo, Francesca Sivori, Mauro Truglio, Daniela Kovacs, Massimo Francalancia, Giovanna D’Agosto, Elisabetta Trento, Grazia Prignano, Arianna Mastrofrancesco, Eva Ruzič-Sabljič, Fulvia Pimpinelli, Enea Gino Di Domenico

**Affiliations:** ^1^ 1Department of Biology and Biotechnology “C. Darwin” Sapienza University of Rome, Rome, Italy; ^2^ Microbiology and Virology, San Gallicano Dermatological Institute, IRCCS, Rome, Italy; ^3^ Laboratory of Cutaneous Physiopathology and Integrated Center of Metabolomics Research, San Gallicano Dermatological Institute, IRCCS, Rome, Italy; ^4^ Institute of Microbiology and Immunology, Faculty of Medicine, University of Ljubljana, Ljubljana, Slovenia

**Keywords:** Lyme disease, *Borrelia afzelii*, skin, erythema migrans, biofilm, *Borrelia garinii*

## Abstract

**Background:**

*Borrelia afzelii* and *Borrelia garinii* are the leading causes of Lyme borreliosis (LB) in Europe. Persistent LB forms may involve biofilms, potentially contributing to antibiotic tolerance.

**Methods:**

Whole genome sequencing (WGS) was conducted on 7 *B. afzelii* and 5 *B. garinii* isolates from erythema migrans skin biopsies. Biofilms were analyzed for extracellular DNA (eDNA) content and biomass. A phenol red metabolic assay assessed the minimum inhibitory concentration (MIC) and minimum biofilm inhibitory concentration (MBIC) of amoxicillin, azithromycin, ceftriaxone, and doxycycline.

**Results:**

Phylogenetic analysis revealed *B. afzelii* and *B. garinii* formed distinct clades, while *B. burgdorferi* B31 clustered separately. Core genome analysis showed 38.9% of genes were shared between *B. afzelii* and *B. garinii*, decreasing to 26.1% with *B. burgdorferi*. The cloud genome expanded from 34.4% to 53.4% with the addition of *B. burgdorferi*. No antimicrobial resistance genes were detected. Surface adhesion gene profiles exhibited significant variation across species, suggesting potential functional differences in host adaptation. *B. afzelii* and *B. garinii* species exhibited biofilms, with biomass correlating significantly with eDNA production. MIC values were 0.25 μg/mL (amoxicillin, ceftriaxone), 0.125 μg/mL (azithromycin), and 0.5 μg/mL (doxycycline), with no significant interspecies differences. However, MBIC values were considerably higher: 2 μg/mL (amoxicillin, azithromycin), 16 μg/mL (ceftriaxone), and 32 μg/mL (doxycycline).

**Conclusions:**

Biofilms in *B. afzelii* and *B. garinii* significantly reduce antibiotic efficacy, particularly ceftriaxone and doxycycline. These *in vitro* findings highlight the need for targeted therapeutic strategies and suggest biofilms may impact treatment outcomes in LB.

## 1 Introduction

Lyme borreliosis (LB) is the most prevalent tick-borne disease in temperate climate zones worldwide. *Borrelia burgdorferi* in North America and *Borrelia afzelii* and *Borrelia garinii* in Europe and Asia are the primary causative agents (Ružić-Sabljić et al., 2008; Steere et al., 2016). Clinical symptoms range from localized skin manifestations (erythema migrans) to systemic involvement, including neuroborreliosis and arthritis. Successful frontline treatments for early LB involve using antibiotics, such as doxycycline, amoxicillin, azithromycin, cefuroxime, and ceftriaxone (Wormser et al., 2000; Eppes and Childs, 2002; Sicklinger et al., 2003). Although standard antibiotic therapy is effective in most cases of early LB, complications arise with persistent forms of the disease that fail to resolve after antibiotic administration (Veinović et al., 2016; Strnad et al., 2023).

Despite appropriate treatment, several reports suggest that more than 10-20% of patients with LB treated for erythema migrans continue to experience symptoms of fatigue, musculoskeletal pain and cognitive impairment. Persistent LB has been a subject of intense research and debate, particularly concerning its pathogenesis and optimal management strategies. Understanding and addressing the challenges of persistent LB is crucial for improving patient outcomes and developing more effective therapeutic approaches (Steere et al., 2016).

An emerging body of evidence points to the role of biofilms in the persistence of LB (Sapi et al., 2019). Biofilms are structured communities of bacterial cells enclosed in a self-produced polymeric matrix that adheres to biological or non-biological surfaces. These complex structures protect bacteria from environmental stresses, including the host’s immune responses and antimicrobial agents. This can result in chronic infection states that are more difficult to eradicate (Di Domenico et al., 2018). Understanding the biofilm-forming capabilities of *Borrelia* species is thus essential for advancing our knowledge of LB persistence and developing more effective therapeutic approaches (Horowitz et al., 2020). The protective biofilm matrix impedes antibiotic penetration and facilitates a microenvironment for bacterial survival and even proliferation under antimicrobial pressure. Consequently, patients with persistent LB often experience prolonged suffering due to the ineffectiveness of standard treatment regimens, underscoring the need for a deeper understanding of biofilm biology in the context of LB.

The propensity of *Borrelia* species to form biofilm and its consequent role in persistent LB is a critical area of research with significant clinical implications. Biofilms are notoriously difficult to disrupt, rendering infections resilient to pharmacological interventions and immune system eradication (Di Domenico et al., 2022). This resilience contributes to the prolonged symptomatology in affected patients, often leading to a decreased quality of life and chronic health issues. Understanding the formation, composition, and resilience of *Borrelia* biofilms is thus essential for advancing the treatment and management of persistent LB.

The study of *Borrelia* biofilms offers profound insights into bacterial behavior and evolution, demonstrating how pathogens adapt to ensure survival within their hosts. These adaptations include gene expression, phenotype, and even genetic composition alterations, driven by the selective pressures imposed by the host immune response and antibiotic treatments. Through such changes, biofilms represent a significant element in bacterial resistance and a focal point for persistent infection, making them a prime target for therapeutic intervention.

The complexity of persistent LB and the role of biofilms require a comprehensive approach, integrating genomic, phenotypic and therapeutic investigations. This study aims to elucidate the genomic and phenotypic characteristics that confer biofilm-forming abilities to *B. afzelii* and *B. garinii*. Additionally, phenotypic assays characterize biofilms and measure antibiotic susceptibility to inform more effective treatments against *Borrelia* infections.

## 2 Materials and methods

### 2.1 Borrelia strains

From the Institute of Microbiology and Immunology, Faculty of Medicine, University of Ljubljana (Ljubljana, Slovenia), 7 *B. afzelii* and 5 *B. garinii* clinical isolates were obtained from skin biopsies of patients with Lyme disease. These patients had typical erythema migrans (EM) diagnosed at the LB Outpatient Clinic of the Department of Infectious Diseases, University Medical Centre Ljubljana, Slovenia; they had not received antibiotics before the examination, underwent a skin biopsy and culture according to standard protocol (Ružić-Sabljić et al., 2008).

Isolated *Borrelia* strains were cultured in the laboratory of Microbiology and Virology, San Gallicano Dermatological Institute, IRCCS, Rome, Italy, in Barbour-Stoner-Kelly H (BSK-H) complete medium, with 6% rabbit serum (Sigma, St Louis, MO). Cultures were incubated in sterile 10 mL glass tubes at 33°C with 5% CO_2_ to reach the mid-log phase (1 x 10^7^ cells/ml). Spirochete viability was monitored daily using a Petroff-Hausser counting chamber and dark-field microscopy.

Ethical approval was obtained from the Central Ethics Committee of I.R.C.C.S. Lazio, Rome (Protocol 6529, 14.05.2021, trial registry number 1536/21), in accordance with the Helsinki Declaration.

### 2.2 Whole-genome analysis

DNA Extraction and Sequencing DNA for whole-genome sequencing (WGS) was extracted using the QIAsymphony DSP Virus/Pathogen Kits (Qiagen, Hilden, Germany), following the manufacturer’s protocols. WGS was performed using the MiSeq platform, and bioinformatics analyses, including short-read quality filtering, assembly, and annotation, were conducted using Bactopia suite v.3.1.0. Assembly quality was assessed using QUAST 5.3.0, while genome completeness was evaluated with BUSCO 5.8.2 against the *borreliaceae_odb12* database. KSNP v4.1 generated the Maximum Likelihood phylogenetic tree based on core SNPs identified across the genome sequences.

Pan-genome analysis used Panaroo v.1.5.1, which clustered genes to identify core and accessory genomes. In addition to the 7 *B. afzelii* and 5 *B. garinii* strains, complete and fully annotated assemblies for *B. burgdorferi* (n=19)*, B. garinii* (n=12), and *B. afzelii* (n=28) were downloaded from NCBI to ensure comprehensive representation in the pan-genome.

Antibiotic resistance genes were predicted using the Comprehensive Antibiotic Resistance Database (CARD) v3.2.8 and the Resistance Gene Identifier (RGI) tool (Alcock et al., 2023). Predictions focused on “perfect” and “strict” matches against high-quality reference sequences, with a 97% identity cutoff for inclusion. Known and candidate *B. burgdorferi* genes implicated in surface adhesion (Antonara et al., 2011; Steere et al., 2016; Robertson et al., 2019; Lemieux et al., 2023) were identified by Blastn searches. Hits with ≥40% coverage and ≥70% identity were considered significant and included in the analysis (Chen et al., 2016). The dendrogram was generated using Python’s scipy hierarchical clustering with Ward’s method.

### 2.3 Antimicrobial agents and experimental design

The tested antibiotics were those recommended for treating LB, such as amoxicillin, azithromycin, ceftriaxone, and doxycycline (Wormser et al., 2000). Stock solutions of antibiotics were prepared in sterile water (1 mg/ml). The antimicrobial agents were diluted in BSK-H medium at concentrations ranging from 0.001 to 16 µg/ml.

### 2.4 Minimum inhibitory concentration (MIC) assessment

Spirochetes were cultured in BSK-H medium for seven days at 33°C to reach the mid-log phase (1 x 10^7^ cells/ml), as determined by enumeration using a Petroff-Hausser counting chamber and dark-field microscopy. A 96-well polystyrene flat-bottom plate was inoculated with a 2-fold dilution series of each antimicrobial agent, prepared in 200 µl of BSK-H medium containing 1 x 10^6^ spirochetes/ml and phenol red (25 µg/ml), as a growth indicator (Robertson et al., 2019). The well plate was covered with parafilm and placed in the incubator at 33°C for seven days. Colorimetric changes were measured due to the acidification of BSK-H medium containing phenol red from the growth of viable spirochetes (Hunfeld et al., 2000; Sapi et al., 2011) to determine antimicrobial susceptibility. Absorbance values at 562 nm (corrected for absorbance at 630 nm) were measured at 0 and 7 days using a Multiskan SkyHigh spectrophotometer (Thermo Fisher Scientific, Ohio, United States). Changes in absorbance were calculated by comparing the absorbance at seven days (At7) with the initial absorbance (At0) for each well (At0 - At7) and then adjusted for changes in the negative control (BSK-H medium without spirochetes). The colorimetric minimum inhibitory concentration (MIC) was defined as the lowest antibiotic concentration at which four replicates’ average decrease in absorbance was ≤25% of the decline observed in the positive control without antibiotics. *B. burgdorferi* strain B31 (ATCC 35210) was included (**Supplementary Table 1**) as a reference strain to facilitate comparison with previously published studies on the *in vitro* susceptibility of Borrelia species (Hunfeld et al., 2000). Each strain was tested in quadruplicate, and the experiments were repeated three times.

### 2.5 Minimal biofilm inhibitory concentration measurements

For biofilm formation, a 96-well plate coated with rat-tail collagen type I (Corning BioCoat Collagen I 96-well flat bottom TC-treated microplate, NY, United States) was inoculated with 200μL of 1 x 10^6^ cell/ml. After incubation at 33°C for seven days, the wells were rinsed with a 0.45% saline solution to eliminate nonadherent cells. Subsequently, 200 μL of BSK-H containing 2-fold serial dilutions of antibiotics with phenol red (25 µg/ml) was added to each well. After five days of incubation, colorimetric changes were measured to determine antimicrobial susceptibility. The colorimetric minimal biofilm inhibitory concentration (MBIC) was the lowest antibiotic concentration at which four replicates’ average decrease in absorbance was ≤25% of the decline observed in the positive control without antibiotics. Each strain was tested in quadruplicate, and experiments were repeated three times.

### 2.6 Assessment of the composition of *B. afzelii* and *B. garinii* biofilms

For biofilms, a 96-well plate coated with rat-tail collagen type I (Corning BioCoat Collagen I 96-well clear flat bottom TC-treated microplate, NY, United States) was inoculated with 200μL of 1 x 10^6^ cell/ml (Sapi et al., 2011). After incubation at 33°C for seven days, the wells were rinsed with 0.45% saline solution to remove nonadherent cells. The resulting cells were stained with 0.01% crystal violet (CV) and incubated at RT for 10 min. Subsequently, 200 μl of 10% acetic acid was added to the wells to extract the stain, and optical density was measured at 595 nm using a Multiskan SkyHigh spectrophotometer (Thermo Fisher Scientific, Ohio, United States) (Timmaraju et al., 2015; Di Domenico et al., 2021; Cavallo et al., 2022).

To quantify extracellular DNA (eDNA), biofilm cultures were resuspended in 100 μL Tris-EDTA (TE) buffer followed by 100 μL freshly made PicoGreen solution (1 μL PicoGreen dye in 199 μL TE buffer). Wells with PicoGreen were incubated for 5 min before measuring the fluorescence intensity (Ex. λ 485 nm, Em. λ 535 nm) using a fluorescence plate reader (Wallace Victor 3, 1420 Multicolor; PerkinElmer). Each analysis was performed on three biological replicates for each strain. Lambda DNA (Invitrogen Molecular Probes) generated a standard curve for each run (Sivori et al., 2022).

### 2.7 Biofilm imaging

Biofilms were grown on μ-Slide (Ibidi, Gräfelfing, Germany) inoculated with 1 x 10^6^ cell/ml in fresh BSK-H medium. After incubation at 33°C for seven days, the wells were rinsed with a 0.45% saline solution to eliminate nonadherent cells. After a brief air-drying step and according to supplier specifications, under controlled conditions to minimize desiccation stress, biofilms were stained using the LIVE/DEAD BacLight Bacterial Viability Kit (Life Technologies, New York, NY, United States), and diluted 1:1000 TOTO-1 staining (Thermo Fisher Scientific, Waltham, MA United States) for the detection of living cells, and eDNA. Viability was assessed immediately after staining to ensure reliable detection of live and dead cell populations. *B. burgdorferi* strain B31 (ATCC 35210) was included (**Supplementary Figure 1**) as a reference strain. The stained biofilms were then visualized with an Axio Observer inverted fluorescence microscope equipped with an Apotome system (Carl Zeiss International, Oberkochen, Germany). Data were analyzed using ZEN 3.2 (Blue Edition) software (Truglio et al., 2024).

### 2.8 Statistical analysis

All variables were summarized with descriptive statistics and tested for normality. When appropriate, continuous variables were compared with Student’s t test or the Mann-Whitney U test. Categorical variables were tested using the χ2 or two-tailed Fisher’s exact test. Correlation analysis was performed using the Pearson coefficient (r). Statistical analyses were performed using SPSS version 21 (SPSS, Inc., Chicago, IL, USA) and GraphPad Prism 10.0 (GraphPad Software, Inc., San Diego, CA, USA). Differences were considered statistically significant for p-values of p < 0.05 (*), p < 0.01 (**), and p < 0.001 (***).

## 3 Results

### 3.1 Genomic diversity and phylogenetic analysis of *B. afzelii* and *B. garinii* isolates

Twelve isolates, including seven *B. afzelii* and five *B. garinii* strains, were collected from the skin biopsy specimens of patients with erythema migrans.

**Figure 1:**
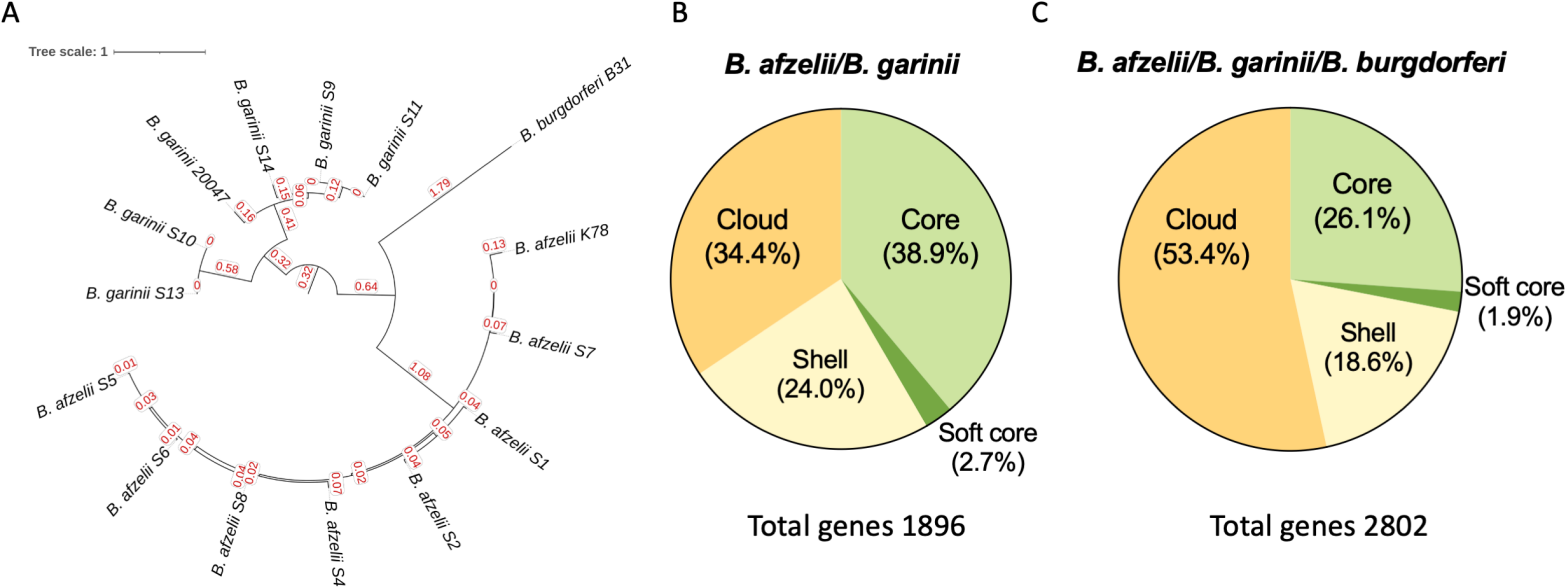
Comparative genomic analysis of *Borrelia* species. **(A)** Maximum-likelihood phylogenetic tree based on single-nucleotide polymorphisms (SNPs) of 7 *B. afzelii*, 5 *B. garinii* clinical isolates, and the *B. burgdorferi* B31, *B. afzelii* K78, *and B. garinii* 20047 used as reference strains. **(B)** Pie charts displaying the percentage of core, soft core, shell and cloud genes shared between *B. afzelii* and *B. garinii* and among **(C)** *B. afzelii*, *B. garinii* and *B. burgdorferi*.

Sequence data for *B. afzelii* and *B. garinii* strains were mapped against *B. burgdorferi* B31 (ASM868v2), *B. afzelii* K78 (ASM96277v1) *and B. garinii* 20047 (ASM381440v1) as reference genome. The maximum likelihood phylogenetic tree based on single-nucleotide polymorphisms (SNPs) (**Figure 1A**) identified two major clades, the *B. afzelii* and *B. garinii* strains, separated by the *B. burgdorferi* B31 strain. The pairwise SNP patristic distance analysis (sum of branch lengths) reveals the genetic divergence among *B. afzelii*, *B. garinii*, and *B. burgdorferi* B31. *B. afzelii* isolates exhibited a minimum SNP distance of 3.061 and a maximum of 3.079 from *B. burgdorferi* B31, while *B. garinii* isolates showed a greater divergence, ranging from 3.671 to 3.733.

As reported in **Figure 1B**, the core genome (genes shared by 99-100% of the strains) accounted for a substantial portion of the genome, comprising 737 genes (38.9%) in the *B. afzelii* and *B. garinii* comparison. This number slightly declined to 733 genes (26.1%) when *B. burgdorferi* was included. The soft-core genome (genes shared by 95-99% of the strains), consisting of genes present in most strains, contained 52 genes (2.7%) without *B. burgdorferi* and 53 genes (1.9%) when it was included. Shell genes, found in 15–95% of strains, represented 455 genes (24.0%) in the *B. afzelii* and *B. garinii* comparison, increasing to 520 genes (18.6%) with *B. burgdorferi*.

Cloud genes, the rarest genetic elements (present in <15% of strains), constituted 652 genes (34.4%) in the two-species comparison but significantly increased to 1496 genes (53.4%) upon including *B. burgdorferi*. Consequently, the total pangenome expanded from 1896 genes for *B. afzelii* and *B. garinii* to 2802 genes when *B. burgdorferi* was added, underscoring its substantial species-specific genetic content (**Figure 1C**).

No statistically significant differences were observed among strain groups in the relative abundances of pan-genome components. However, the marked increase in cloud genes upon including *B. burgdorferi* (from 34.4% to 53.4%) highlights its considerable genomic distinctiveness.

The CARD analysis predicted the absence of AMR genes in all *B. afzelii* and *B. garinii* isolates.

### 3.2 Analysis of surface adhesion genes

**Figure 2:**
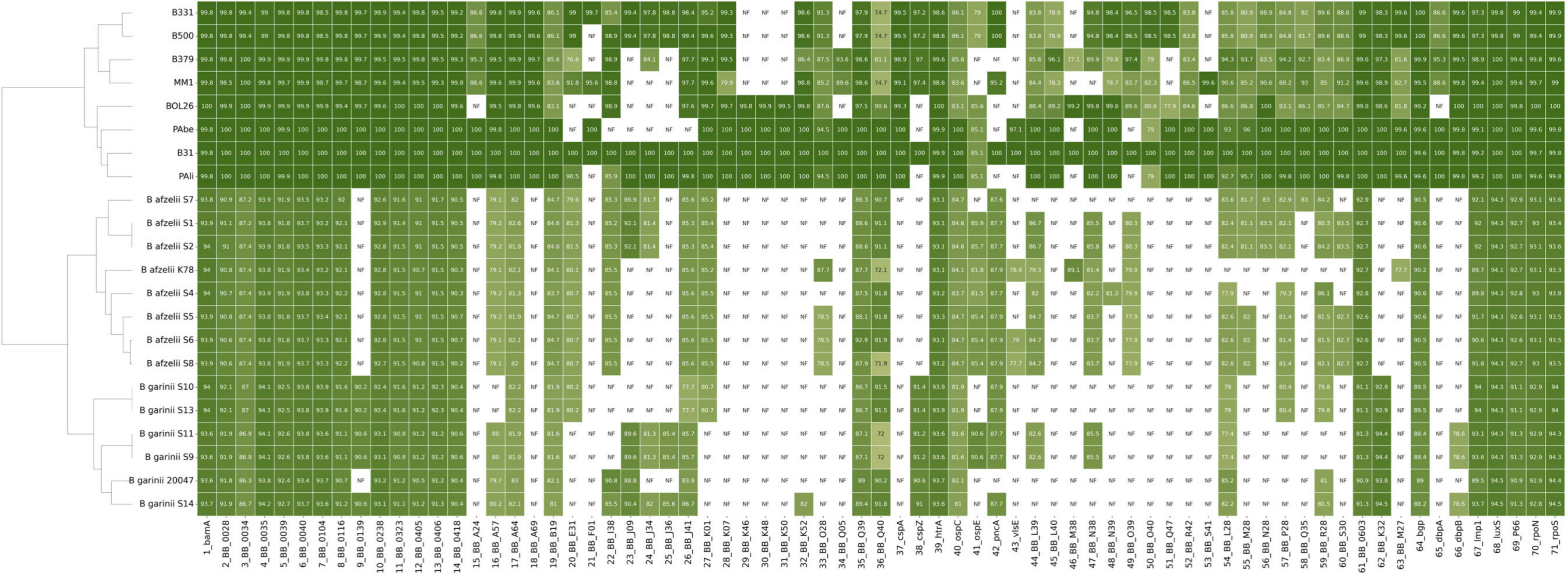
Heatmap of surface adhesion gene distribution across *Borrelia* species. The heatmap illustrates the presence (shaded cells) or absence (white cells) of surface adhesion genes among *B. burgdorferi* (B331, B500, B379, MM1, BOL26, PAbe, B31, and Pali), *B. afzelii*, and *B. garinii*. The intensity of the shading correlates with the sequence identity of the indicated gene compared to the *B. burgdorferi* B31 reference strain. *B. afzelii* K78, and *B. garinii* 20047 were included as quality controls. Hierarchical clustering on the left delineates phylogenetic relationships among the strains. Each row represents a different *Borrelia* strain and each column corresponds to a specific gene.

Surface adhesion is a crucial step in the establishment of microbial communities (Antonara et al., 2011). To identify genetic elements involved in surface adhesion, pan-genome elements from each sequenced isolate were annotated using BLAST and compared with the *B. burgdorferi* B31 reference genome (**Figure 2**). Based on the presence or absence of adhesion-related genes, hierarchical clustering revealed significant (P<0.0001) interspecies differences. *B. burgdorferi* exhibited a significantly higher number of annotated adhesion-associated genes compared to *B. afzelii* (P<0.0001) and *B. garinii* (P<0.0001). Notably, *B. afzelii* and *B. garinii* displayed a comparable gene count. The heatmap visualization illustrates the distribution of these genes, with *B. burgdorferi* forming a distinct cluster enriched in adhesion-related loci. The substantial absence of several adhesion genes in *B. afzelii* and *B. garinii* underscores their genetic divergence from *B. burgdorferi*, suggesting potential differences in their capacity to initiate surface attachment.

### 3.3 Analysis of biofilm structure

**Figure 3:**
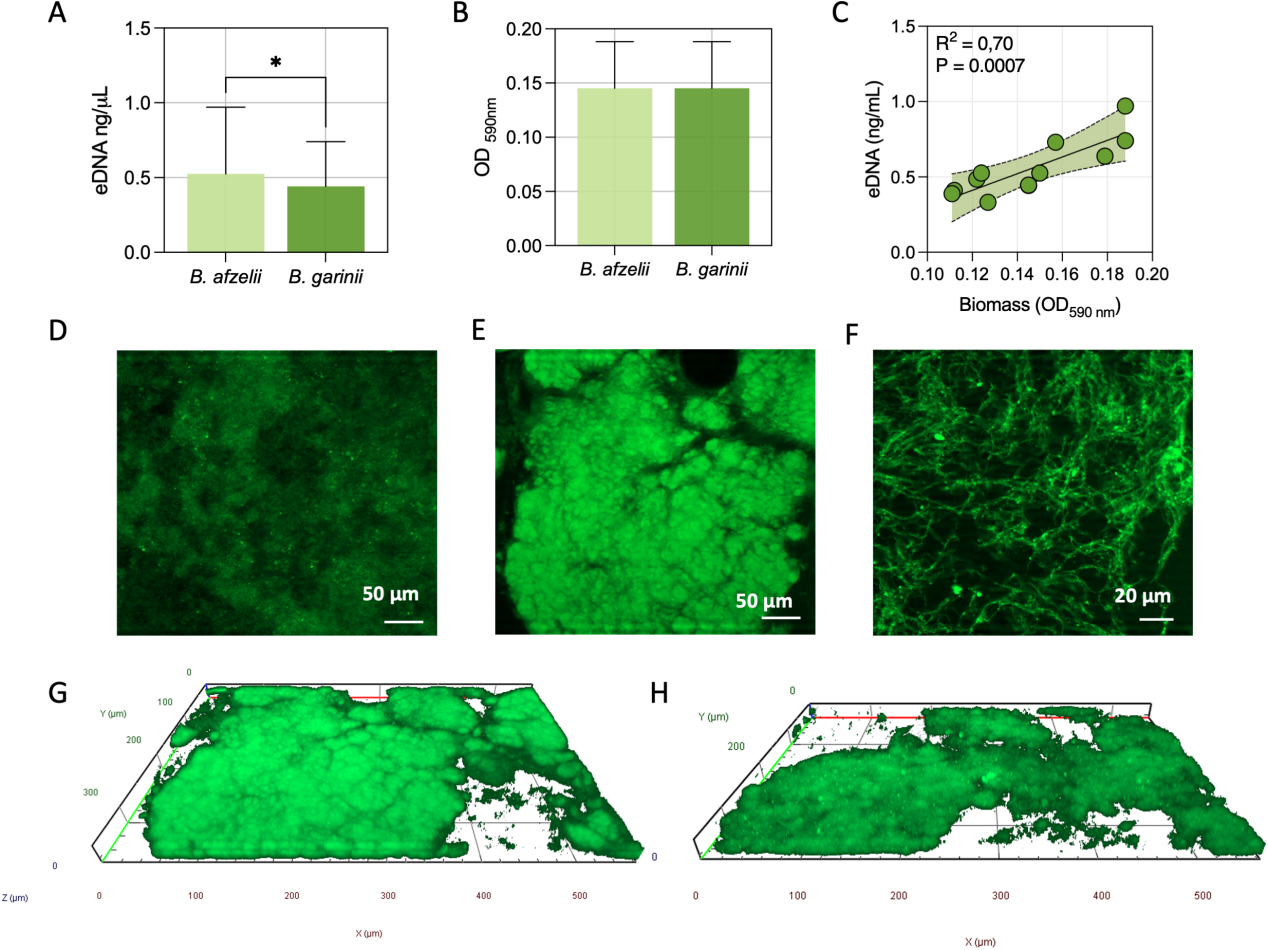
Quantitative and structural analysis of biofilms in *B. afzelii* and *B. garinii*. **(A)** Extracellular DNA (eDNA) quantification in biofilms. **(B)** Biomass measurement using Crystal Violet staining. **(C)** Pearson’s rank correlation analysis between eDNA content and biomass across strains. **(D)** Representative fluorescence microscopy images of TOTO-1-stained eDNA in biofilms. Fluorescence microscopy images of the central **(E)** and peripheral **(F)** regions of biofilms at different magnifications, stained with the Live/Dead BacLight bacterial viability kit. Computerized 3D reconstructions of biofilms formed by *B. afzelii* **(G)** and *B. garinii* **(H)**, with X, Y, and Z axes in μm. Statistical significance was assessed using the Mann-Whitney U test. *, p < 0.05.

The distribution of genes for surface-exposed lipoproteins and adhesion factors was comparable between *B. afzelii* and *B. garinii*. Extracellular DNA (eDNA), critical for initial surface attachment, and biomass production, were phenotypically characterized. *B. afzelii* exhibited significantly higher eDNA levels (0.62 ± 0.18 ng/µl) compared to *B. garinii* (0.48 ± 0.15 ng/µl, P = 0.016) (**Figure 3A**). However, no significant differences were observed in biomass production, with *B. afzelii* (OD_590_ = 0.14 ± 0.03) and *B. garinii* (OD_590_ = 0.15 ± 0.03) showing similar values (**Figure 3B**). Given the relatively low variability in eDNA and biomass among isolates, we assessed whether these phenotypes were coregulated or functioned independently. Pearson’s correlation analysis revealed a significant positive association between biomass and eDNA levels (P = 0.0007), suggesting a coordinated regulatory mechanism (**Figure 3C**).

Fluorescence microscopy further analyzed the biofilm composition and spatial organization (**Figure 3**). The BacLight Live/Dead staining highlighted dense cellular aggregates, confirming the formation of a biofilm (**Figure 3D**). Higher-magnification imaging of the peripheral regions revealed interconnected spirochetal networks (**Figure 3E**). Additionally, TOTO-1 staining demonstrated the presence of eDNA within the matrix (**Figure 3F**).

Three-dimensional reconstruction further confirmed that *B. afzelii* (**Figure 3G**) and *B. garinii* (**Figure 3H**) formed densely packed biofilms with a thickness ranging from 35 to 45 µm (**Figures 3G, H**).

### 3.4 Antibiotic susceptibility of biofilm-producing isolates

All strains demonstrated densely packed biofilms. To assess whether biomass production conferred increased antibiotic resistance, minimum inhibitory concentration (MIC) and minimal biofilm inhibitory concentration (MBIC) for amoxicillin, azithromycin, ceftriaxone and doxycycline were compared (**Table 1**, **Supplementary Table 2**). Specifically, the MIC values for amoxicillin, azithromycin, ceftriaxone and doxycycline were 0.25 μg/mL (0.064–1.0 μg/mL), 0.125 μg/mL (0.064–0.25 μg/mL), 0.25 μg/mL (0.064–0.25 μg/mL), and 0.5 μg/mL (0.25-2 μg/mL), with not significantly differences between *B. afzelii* or *B. garinii* isolates (**Table 1**). The MBIC of the *Borrelia* isolates gave profiles that significantly (P < 0.001) differed from those gathered by MIC for each antibiotic (**Table 1**). Notably, median MBIC values were 2 μg/mL (0.5-8 μg/mL) for amoxicillin, 2 μg/mL (0.25-8 μg/mL) for azithromycin, 16 μg/mL (1->16 μg/mL) for ceftriaxone and >16 μg/mL (4->16 μg/mL). The MBEC/MIC ratio, which indicates the fold increase in the antimicrobial dose needed to kill *Borrelia* cells in biofilms compared to planktonic growth, was used to quantify the antibiotic tolerance (**Table 1**). The maximum MBEC/MIC value was 64.0, reported for ceftriaxone and >32 for doxycycline, followed by 16.0 observed for azithromycin and 8 for amoxicillin.

**Table 1:** Antimicrobial susceptibility profiles of *Borrelia afzelii* and *Borrelia garinii* in planktonic and biofilm growth phases.

Antibiotic	MIC	MBIC	MBIC/MIC	P
	Median (Range)	Median (Range)	Median	
Amoxicillin (tot)	0.25 (0.064 – 0.5)	2 (0.5 - 8)	8	<0,0001
*B. afzelii*	0.25 (0.125 – 0.5)	2 (1 – 8)		0.0006
*B. garinii*	0.25 (0.064 – 0.5)	2 (0.5– 8)		0.0159
Azithromycin (tot)	0.125 (0.064 – 0.5)	2 (0.25 – 8)	16	<0,0001
*B. afzelii*	0.125 (0,064 – 0.5)	2 (0.25 – 2)		0.0017
*B. garinii*	0.25 (0.064 – 0.5)	4 (0.25 – 8)		0.0079
Ceftriaxone (tot)	0.25 (0.064 – 0.25)	16 (1 – 32)	64	<0,0001
*B. afzelii*	0.25 (0.064 – 0.25)	16 (2 - 32)		0.0006
*B. garinii*	0.25 (0.125 – 0.25)	16 (1 - 32)		0.0079
Doxycycline (tot)	0.5 (0.25 - 2)	32 (4 - 32)	64	<0,0001
*B. afzelii*	0.5 (0.25 – 2)	32 (8 - 32)		0.0006
*B. garinii*	0.5 (0.25 – 1)	32 (4 - 32)		0.0079

Minimum Inhibitory Concentrations (MICs, µg/ml) were determined for planktonic spirochetes cultured in BSK-H medium following standard microdilution protocols. Minimum Biofilm Inhibitory Concentrations (MBICs, µg/ml) were determined for seven-day mature biofilms. Values represent geometric means from triplicate experiments. Tot (total) represents combined susceptibility data for both species; P indicates the statistical significance of the difference in MIC or MBIC values between *B. afzelii* and *B. garinii* (Mann-Whitney U test).

The MBIC of the *Borrelia* isolates significantly differed (P < 0.001) from the MIC profiles for each antibiotic tested (**Table 1**). Notably, the median MBIC values were 2 μg/mL (ranging from 0.5 to 8 μg/mL) for amoxicillin, 2 μg/mL (0.25 to 8 μg/mL) for azithromycin, 16 μg/mL (1 to 32 μg/mL) for ceftriaxone, and 32 μg/mL (4 to 32 μg/mL) for doxycycline. The MBEC/MIC ratio, which quantifies the fold increase in the antimicrobial dose required to eliminate *Borrelia* cells in biofilms compared to planktonic cells, revealed significant antibiotic tolerance (**Table 1**). The highest MBEC/MIC value was 64.0 for ceftriaxone and doxycycline, 16.0 for azithromycin, and 8.0 for amoxicillin.

## 4 Discussion

The results of this study reveal substantial portions of core genes within these *Borrelia* species, highlighting significant conservation across the genomes of *B. afzelii* (44.5%) and *B. garinii* (43.4%), compared to *B. burgdorferi* (52.5%). Moreover, the core genome accounts for 9.4% of the genes between *B. afzelii* and *B. garinii* and an overall 7.2% of conservation among the three *Borrelia* species. The core genome conservation observed in our study is consistent with findings from previous research, which also reported a high degree of genetic stability among *Borrelia* species (Margos et al., 2008; Schutzer et al., 2011; Lemieux et al., 2023). This level of core genome conservation suggests essential functions are preserved to maintain the pathogenicity and adaptability of these bacteria across different hosts and environments. Interestingly, the low percentage of core genes shared between *B. afzelii* and *B. garinii*, and even lower among all three species, indicates a significant genetic differentiation. This differentiation may play a role in shaping the ecological adaptation of each *Borrelia* species, with potential implications for pathogenicity. Previous studies have highlighted patterns of core genome conservation and genetic divergence, underscoring the evolutionary pressures that shape these genomes (Qiu et al., 2004; Margos et al., 2008). Our findings emphasize the importance of considering both conserved and variable genomic elements when studying the biology and epidemiology of *Borrelia* species. Indeed, the substantial conservation of core genes supports the development of broad-spectrum diagnostic tools and antimicrobial treatments. At the same time, the observed genetic differences can inform species-specific strategies for managing Lyme disease (Gwynne et al., 2023). The analysis of COG categories demonstrated notable differences in functional gene distributions among the *Borrelia* species. *B. afzelii* exhibited fewer genes related to nucleotide transport and metabolism than *B. garinii* and *B. burgdorferi*, indicating species-specific metabolic adaptations. Conversely, *B. afzelii* showed a higher number of genes involved in replication, recombination, and repair than *B. burgdorferi*, which may contribute to its enhanced ability to survive under stressful conditions (Strnad et al., 2023). These differences may correspond to specific changes each species has undergone to adapt to its respective ecological niche. For instance, gene variations related to metabolism, replication and repair mechanisms could reflect adaptations to different host environments or transmission cycles. For example, differences in nutrient acquisition and metabolic pathways may be crucial for survival in various host tissues or during different life cycle stages, including transmission by tick vectors (Strnad et al., 2023; Čorak et al., 2023). Additionally, the presence or absence of genes related to stress response and DNA repair mechanisms may indicate how each species copes with the host immune response or environmental stressors, influencing their persistence and virulence (Caimano et al., 2007).

Our phenotypic assays confirmed that *B. afzelii* and *B. garinii* form biofilms, and the biomass was significantly correlated with eDNA production. This suggests that eDNA may play a role in maintaining the structural integrity and resilience of these biofilms and may represent a potential therapeutic target to disrupt biomass integrity and combat persistent infections (Sharma and Rajpurohit, 2024).

The most striking finding of this study pertains to the MBICs compared to the MICs. The MIC values for amoxicillin, azithromycin, ceftriaxone, and doxycycline were consistent with those reported in the literature for planktonic *Borrelia* species, demonstrating high susceptibility (Hunfeld et al., 2000). This susceptibility was corroborated by WGS results, which revealed the absence of antimicrobial resistance genes (ARGs) in *B. afzelii* and *B. garinii* isolates. This aligns with previous studies reporting a substantial absence of ARGs in *Borrelia* species (Papp et al., 2023). The lack of ARGs in our *Borrelia* isolates is particularly significant considering the varying antibiotic pressures in different host environments. While wild animals are less likely to encounter substantial antibiotic pressure, domestic animals and humans frequently undergo antibiotic treatments. In humans, many infections are treated with antibiotics, leading to the potential acquisition of ARGs. However, the likelihood of these bacteria re-entering a tick vector is low. In contrast, domestic animals, especially farm animals, present a greater risk for the spread of ARGs due to prevalent antibiotic use. These bacteria can then be transmitted by vectors such as ticks, which may go undetected in these settings. Our findings suggest that *B. afzelii* and *B. garinii* may not be under significant antibiotic pressure in their typical hosts, which might explain the absence of ARGs. This absence could also reflect effective management practices in the environments from which these strains were isolated. These results underscore the importance of continued surveillance and responsible antibiotic use in both human and animal health sectors to prevent the emergence and spread of antibiotic resistance among *Borrelia* isolates (Papp et al., 2023).

The MBIC values were significantly higher compared to MICs, highlighting the reduced efficacy of these antibiotics against biofilm-associated bacteria. For instance, the MBIC for ceftriaxone was 16 µg/mL, a 64-fold increase from its MIC of 0.25 µg/mL. Similarly, doxycycline showed an MBIC of 32 µg/mL, indicating a 64-fold increase from its MIC of 0.5 µg/mL. These findings align with previous studies that have demonstrated the inherent resistance of biofilms to antibiotic treatment in *B. burgdorferi* (Sapi et al., 2019). In particular, in stationary-phase cultures (7–15 days old), *B. burgdorferi* within biofilms exhibited increased survival following antibiotic treatment compared to the motile spirochetal form (Feng et al., 2014, 2015; Caskey and Embers, 2015; Sharma et al., 2019). The significantly higher MBIC values suggest that standard antibiotic treatments may be insufficient, potentially leading to persistent infections and treatment failures (Di Domenico et al., 2022). The increased MBIC values may imply the need for alternative therapeutic strategies that can effectively penetrate or disrupt the biomass matrix. The absence of detectable ARGs in the studied isolates suggests that the observed ability to escape antibiotic treatment may be predominantly due to the formation of a biofilm rather than genetic resistance mechanisms. Suspecting these *in vitro* findings proceed equivalently *in vivo* emphasizes the importance of critical discussion regarding developing therapeutic strategies to disrupt biofilms or prevent their formation effectively. The higher MIC values reported in this study, relative to those of previous investigations (Feng et al., 2014; Li et al., 2023), may be partly explained by the use of a higher initial inoculum (1 × 10^6^ vs. 1 × 10^5^ spirochetes/mL). Inoculum size is a well-recognized determinant of antimicrobial susceptibility, with increased cell density often associated with elevated MICs. In the absence of interpretive breakpoints for *Borrelia* spp. from CLSI or EUCAST, we adopted a protocol utilizing a 1 × 10^6^ spirochetes per mL inoculum, as previously described (Hunfeld et al., 2000). These methodological discrepancies underscore the need for standardized guidelines in susceptibility testing for *Borrelia* species.

Despite the significant findings, this study has several limitations. The sample size was relatively small, with 12 isolates (7 *B. afzelii* and 5 *B. garinii*) analyzed. This limited sample size may not fully capture the genetic and phenotypic diversity of *B. afzelii* and *B. garinii* species in broader populations. Moreover, a further limitation of this study lies in the absence of systematically integrated clinical metadata linked to the *Borrelia* isolates analyzed. While all strains were derived from patients with clinically confirmed erythema migrans, no additional patient-level variables, such as treatment history, symptom chronology, immune status, or post-treatment outcomes, were available for correlation. This lack of phenotypic linkage limits our ability to assess the clinical relevance of specific genomic or biofilm-associated traits, particularly to disease chronicity, treatment response, and the potential contribution of biofilms to therapeutic failure. Integrating microbiological findings with longitudinal clinical datasets will be essential in future studies to determine whether phenotypic features observed *in vitro*, such as elevated MBICs or enhanced eDNA production, translate into differential clinical trajectories. Such integration would also enable more specific investigations into host-pathogen dynamics, including the influence of host immune factors on *Borrelia* persistence and the stability of biofilms *in vivo*. The study focused on isolates from skin biopsy specimens of patients with erythema migrans, which may not represent the full spectrum of clinical manifestations of LB, including neuroborreliosis and Lyme arthritis. While the study provides valuable insights into biofilm formation and antibiotic resistance, it did not explore the *in vivo* relevance of these mechanisms. Interpreting the role of biofilms in *Borrelia* persistence remains controversial, particularly regarding the long-term survival of viable bacteria post-antibiotic treatment. While *in vitro* studies suggest that *Borrelia* can adopt morphotypes like round bodies and biofilm aggregates, *in vivo* evidence is limited. In a mouse model, Bockenstedt et al. demonstrated that although *B. burgdorferi* DNA and antigens persisted post-treatment, no viable spirochetes were recoverable. Xenodiagnostic studies further confirmed the inability of treated mice to transmit the infection to naïve hosts via ticks or tissue transplants (Bockenstedt et al., 2012). These findings suggest that bacterial DNA detection does not equate to ongoing infection, emphasizing the need for cautious interpretation of *in vitro* biofilm-related findings in a clinical context. Moreover, the clinical implications of biofilms in persistent LB require further investigation through *in vivo* studies by incorporating larger, more diverse cohorts and exploring the interactions between *Borrelia*, host factors, and the environment.

In conclusion, our study highlights the remarkable ability of *B. afzelii* and *B. garinii* to form biofilms, which significantly impede the efficacy of antibiotics. These *in vitro* findings underscore the critical attitude for distinct therapeutic approaches targeting biofilm-associated infections, as is anticipated in LB. Future research should focus on identifying compounds that can disrupt biofilm integrity or enhance the penetration of antibiotics, thereby improving treatment outcomes for patients with persistent infections, as is assumed in LB.

## Data Availability

The datasets presented in this study can be found in online repositories. The names of the repository/repositories and accession number(s) can be found below: https://www.ebi.ac.uk/ena, PRJEB86142.
