# Supplementary figures and images for: Genomic characterization and antibiotic susceptibility of biofilm-forming Borrelia afzelii and Borrelia garinii from patients with erythema migrans
Source: Front Cell Infect Microbiol. 2025 Jul 7;15:1619660. doi: 10.3389/fcimb.2025.1619660 (PMC12277364; doi:10.3389/fcimb.2025.1619660)

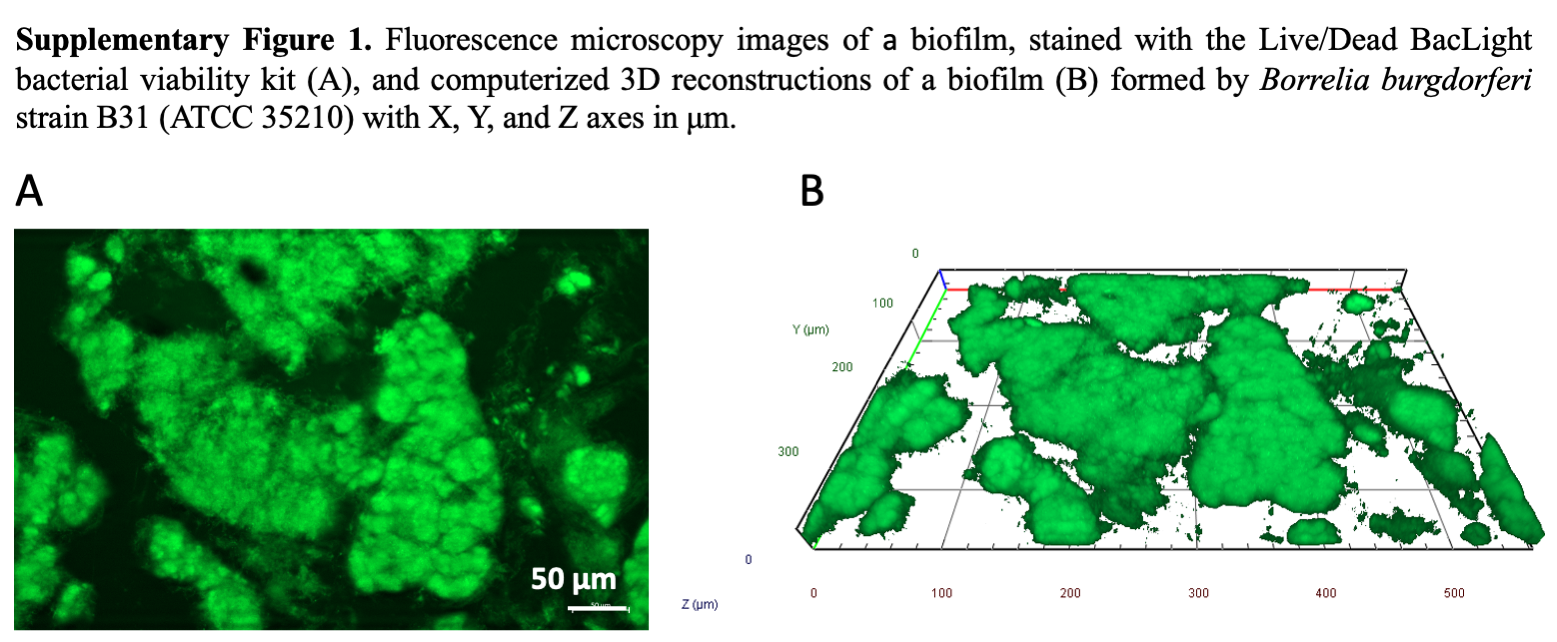

Supplement: Supplementary file 1 [file Image1.tiff]
