# Supplementary material for: Genomic characterization and antibiotic susceptibility of biofilm-forming Borrelia afzelii and Borrelia garinii from patients with erythema migrans
Source: Front Cell Infect Microbiol. 2025 Jul 7;15:1619660. doi: 10.3389/fcimb.2025.1619660 (PMC12277364; doi:10.3389/fcimb.2025.1619660)
Supplement: Supplementary file 2 [file Table1.pdf]

Table 1. Minimum Inhibitory Concentrations (MICs) (μg/ml) of amoxicillin, azithromycin, ceftriaxone, and doxycycline against *Borrelia burgdorferi* strain B31 (ATCC 35210). MIC values refer to planktonic spirochetes cultured under standard conditions.

Antibiotic	MIC (μg/ml)
**Amoxicillin**	0.5
**Azithromycin**	0.03
**Ceftriaxone**	0.064
**Doxycycline**	0.25
