# Supplementary material for: Genomic characterization and antibiotic susceptibility of biofilm-forming Borrelia afzelii and Borrelia garinii from patients with erythema migrans
Source: Front Cell Infect Microbiol. 2025 Jul 7;15:1619660. doi: 10.3389/fcimb.2025.1619660 (PMC12277364; doi:10.3389/fcimb.2025.1619660)
Supplement: Supplementary file 3 [file Table2.pdf]

Table 1. Antimicrobial susceptibility profiles of clinical *Borrelia afzelii* and *Borrelia garinii* isolates in planktonic and biofilm growth phases. Left panel: Minimum Inhibitory Concentrations (MICs, µg/ml) determined for planktonic spirochetes using standard microdilution methodology in BSK-H medium. Right panel: Minimum Biofilm Inhibitory Concentrations (MBICs, µg/ml) determined for seven-day mature biofilms using modified biofilm susceptibility protocols. Values represent individual strain determinations across four antimicrobial agents: amoxicillin, azithromycin, ceftriaxone, and doxycycline.

Minimum Inhibitory Concentrations (MICs, µg/ml)				
**Strain**	**Amoxicillin**	**Azithromycin**	**Ceftriaxone**	**Doxycycline**
B. afzelii-01	0.125	0.125	0.25	0.5
B. afzelii-02	0.125	0.125	0.25	0.5
B. afzelii-03	0.25	0.125	0.25	0.5
B. afzelii-04	0.125	0.125	0.25	0.5
B. afzelii-05	0.5	0.5	0.25	2
B. afzelii-06	0.25	0.25	0.064	0.25
B. afzelii-07	0.25	0.064	0.125	1
B. garinii-01	0.064	0.25	0.125	0.5
B. garinii-02	0.25	0.25	0.25	0.5
B. garinii-03	0.25	0.125	0.125	1
B. garinii-04	0.25	0.064	0.25	0.25
B. garinii-05	0.5	0.5	0.25	1

Minimum Biofilm Inhibitory Concentrations (MBICs, µg/ml)				
**Strain**	**Amoxicillin**	**Azithromycin**	**Ceftriaxone**	**Doxycycline**
B. afzelii-01	2	2	16	16
B. afzelii-02	1	2	2	8
B. afzelii-03	2	2	4	32
B. afzelii-04	1	2	8	32
B. afzelii-05	2	2	16	32
B. afzelii-06	8	2	16	16
B. afzelii-07	2	0.25	32	32
B. garinii-01	0.5	2	1	16
B. garinii-02	2	4	16	32
B. garinii-03	2	4	32	32
B. garinii-04	2	0.25	16	4
B. garinii-05	8	8	16	32
